# Flavonoid biosynthesis and Arabidopsis genetics: more good music

**DOI:** 10.1093/jxb/erw050

**Published:** 2016-02-19

**Authors:** Anton R. Schäffner

**Affiliations:** Institute of Biochemical Plant Pathology, Helmholtz Zentrum München, German Research Center for Environmental Health (GmbH), Ingolstädter Landstr. 1, 85764 Neuherberg, Germany

**Keywords:** *Arabidopsisthaliana*, flavonoid, flavonol glucosyltransferase, glycoside hydrolase-type, natural variation, whole-genome association mapping.

**In this issue of *Journal of Experimental Botany* (pages 1505–1517), Ishihara *et al*. report the identification of a gene responsible for the production of flavonol 3-*O*-gentiobioside 7-*O*-rhamnosides by elegantly tickling the ivories of Arabidopsis genetics and genetic resources combined with straightforward metabolite analysis: it is a model case of functional evaluation.**

Flavonoids are secondary metabolites derived from the phenylpropanoid biosynthetic pathway that occur in a huge number and variety in plants. They have been implicated in diverse processes, including pigmentation, redox and UV protection, plant–microbe interactions, development and regulation of auxin transport (Winkel-Shirley, 2006; Yonekura-Sakakibara *et al.*, 2008; Kuhn *et al.*, 2011; Peer *et al.*, 2011; Grunewald *et al.*, 2012; Buer *et al.*, 2013; Emiliani *et al.*, 2013; Saito *et al.*, 2013; Yin *et al.*, 2014; Ishihara *et al.*, 2016).

Genetics and flavonoid biosynthesis were already successfully engaged on the verge of Arabidopsis becoming the plant model organism. The *transparent testa* (*tt*) mutant loci, which affect the biosynthesis of flavonoids, defined easily scorable genetic markers due to the loss of seed coat pigmentation. Their molecular identification established many crucial steps in the biosynthesis of the flavonoid core structure (Koornneef *et al.*, 1983; Shirley *et al.*, 1995; Winkel-Shirley, 2006; Saito *et al.*, 2013). This core is formed by a phenyl ring condensed with an oxygen-containing heterocycle in different oxidation states which is further substituted at different positions with another phenyl side group. These variable cores constitute different flavonoid classes occurring in plants and within a given plant species. However, only the decoration of these aglycones with various carbohydrate side chains and further chemical modification provides the full flavonoid range.

Again, genetics in combination with biochemistry, metabolite analyses and, in particular, gene co-expression patterns led to the identification of several UDP-carbohydrate-dependent glycosyltransferases (UGTs) conjugating flavonoids with different carbohydrates at different positions (Jones *et al.*, 2003; Yonekura-Sakakibara *et al.*, 2008, 2012). Nevertheless, there are still unresolved cases, one of them being the production of the Arabidopsis accession-specific flavonol 3-*O*-gentiobioside 7-*O*-rhamnosides (F3GG7Rs), comprising the flavonols kaempferol, quercetin or isorhamnetin with the specific carbohydrate decoration 3GG7R, which are most probably derived through glucosylation from flavonol 3-*O*-glucoside 7-*O*-rhamnoside (F3G7R) precursors.

## A novel flavonol glucosyltransferase

Ishihara *et al.* (2016) have now identified a gene responsible for this final step in the production of F3GG7Rs. Previous reports that the accessions L*er* and Nö-0, but not Col-0 and Cvi, contain F3GG7Rs were extended to a collection of 81 accessions, of which just half were F3GG7R-producers. The L*er* F3GG7R trait was inherited in a dominant manner in a cross with the F3GG7R-lacking Col-0. Linkage analysis using an F3GG7R-metabotyped, 95-member-sized L*er* × Col recombinant inbred (RI) population as well as recombination events within the originally identified interval using a further 200 additional RI lines eventually revealed the locus responsible in a small 87kb region on chromosome 1. No obvious candidate, such as a *UGT* gene, was located in that interval; however, genome-wide association mapping of the F3GG7R metabotype of the 81 accessions confirmed the RI linkage mapping and eventually identified a single-nucleotide polymorphism leading to a premature stop codon in the Col-0 allele of *BETA GLUCOSIDASE* 6 (*BGLU6*). In contrast, L*er* contains a fully functional *BGLU6* gene and F3GG7R production was generally associated with functional *BGLU6* alleles.

Transcriptional co-expression analysis has been shown to be a valuable tool in the study of flavonol biosynthesis (see above); thus, the association of *BGLU6* with this pathway further supported its likely involvement in F3GG7R biosynthesis. Nevertheless, this finding is remarkable at first sight, since the underlying expression data were primarily derived from the F3GG7R-deficient Col-0 harbouring only the *BGLU6* pseudogene. However, the promoter sequences of the L*er* and Col-0 alleles are highly similar and thus transcriptional co-regulation was not affected by either functional (L*er*) or non-functional (Col) gene transcripts. On the other hand, Ishihara *et al.* (2016) point out based on published RNA-Seq data that the expression level of functional *BGLU6* alleles was about twofold higher than the transcription from *BGLU6* alleles leading to transcripts harbouring the premature stop codon. The reason for this negative impact on the abundance of the non-functional transcript (either transcription or stability of the mRNA) is not clear, but it may be an interesting future issue in relation to the pseudogenization of gene copies.

After this genetic free-form jazz, the scales were still completed successfully: luckily, *BGLU6*-targeting insertion lines in two F3GG7R-accumulating accessions, L*er* and Ws-4, were available and both led to the loss of F3GG7R production. Conversely, the F3GG7R-deficient Col-0 gained the ability to synthesize F3GG7R after genetic transformation with a functional L*er BGLU6* gene fragment.

## Genetics leading the way

Biochemical proof by an *in vitro* enzymatic activity test could not be provided by Ishihara *et al.* (2016), since expression of the recombinant BGLU6 protein failed in several systems. However, genetics has provided overwhelming evidence that BGLU6 is indeed responsible for F3GG7R formation. This not only adds another piece of information about the complex formation of flavonol glycosides in Arabidopsis, but also provides strong evidence that acyl-carbohydrates utilized as sugar donors by beta-glucosidases, such as the putative beta-glucosidase BGLU6, are involved in flavonol glycosylation in addition to the well-known UDP-carbohydrate donors used by UGTs. This extends recent reports on beta-glycosidases being involved in Arabidopsis anthocyanin glycosylation (Miyahara *et al.*, 2013).

Nevertheless, the identification of this new molecular player being responsible for producing the accession-specific F3GG7R flavonol glycosides could not provide clues to a specific physiological or ecological role. The same is mostly true for the plethora of specifically decorated flavonoids. Most probably, only genetics will be able to lead the way to unraveling such functional relations between specific flavonoid glycosides and particular processes and functions (Yin *et al.*, 2014). More music expected.
